# The Impact of Starvation on the Microbiome and Gut-Brain Interaction in Anorexia Nervosa

**DOI:** 10.3389/fendo.2019.00041

**Published:** 2019-02-12

**Authors:** Jochen Seitz, Meriem Belheouane, Nina Schulz, Astrid Dempfle, John F. Baines, Beate Herpertz-Dahlmann

**Affiliations:** ^1^Department of Child and Adolescent Psychiatry, Psychotherapy and Psychosomatics, University Hospital, RWTH University Aachen, Aachen, Germany; ^2^Institute for Experimental Medicine, Kiel University and Max Planck Institute for Evolutionary Biology, Plön, Germany; ^3^Institute of Medical Informatics and Statistics, Kiel University, Kiel, Germany

**Keywords:** anorexia nervosa, microbiome, inflammation, autoimmune disease, gut-brain axis, nutrition, probiotics, prebiotics

## Abstract

Interactions between the gut microbiome and the brain are of increasing interest to both researchers and clinicians. Evidence is mounting on the causal role of an altered gut microbiome in inflammatory diseases such as arthritis, inflammatory bowel disease, obesity and diabetes, and psychiatric diseases like anxiety and depression. Mechanisms include altered energy harvest from food, hormonal changes, increased gut permeability, inflammation, immune response, and a direct influence on the brain and behavior. Anorexia nervosa (AN) is the third most common disease in adolescence and exacts a high burden on patients and caregivers. It often becomes chronic and has the highest mortality of all psychiatric diseases. As AN is characterized by nutritional restrictions, weight loss, and severe behavioral symptoms including weight phobia, comorbid anxiety and depression, accompanied by endocrine alterations, increased inflammation, and immune response, exploring the role of the gut microbiome is crucial. Here, we present an overview of the potential mechanisms of interaction between the gut microbiome, the host and particularly the brain in AN and summarize the initial findings of microbiome research on AN. We conclude by identifying future research directions and potential therapeutic approaches, including nutritional interventions, probiotics, prebiotics and food supplements, that could become important additions to current AN therapy.

## Introduction

Anorexia nervosa (AN) is one of the most common chronic illnesses in adolescence, with a lifetime prevalence of 1–4% in 12–18-year-old girls in Europe (1). Recent studies report incidence rates of 100–200/100,000 person-years in 15–19-year-old females, which is similar to that of type 1 diabetes (2, 3). In Germany and the UK, the number of hospitalized children and adolescents has risen substantially during the last decade (4), and the age of onset has decreased (5). After a relatively brief window of treatment opportunity, the starvation process in AN is often self-perpetuating (6). AN has a high risk of becoming chronic and has the highest mortality of all psychiatric disorders (7, 8). However, in the early stages of the disorder, no indicator currently exists that enables predicting the course and outcome of the disorder. In an 18-year follow-up study, 25% of the subjects with previous adolescent AN were unemployed because of a mental disorder (9). Psychiatric and somatic comorbidity, including depression, anxiety and starvation-induced somatic changes such as endocrine dysfunctions and osteoporosis is high, and therefore further impedes a favorable outcome. A multimodal treatment approach involving weight rehabilitation and psychotherapy is the current state-of-the-art approach, but underlying pathophysiology and proliferating factors are not well understood.

**Figure 1:**
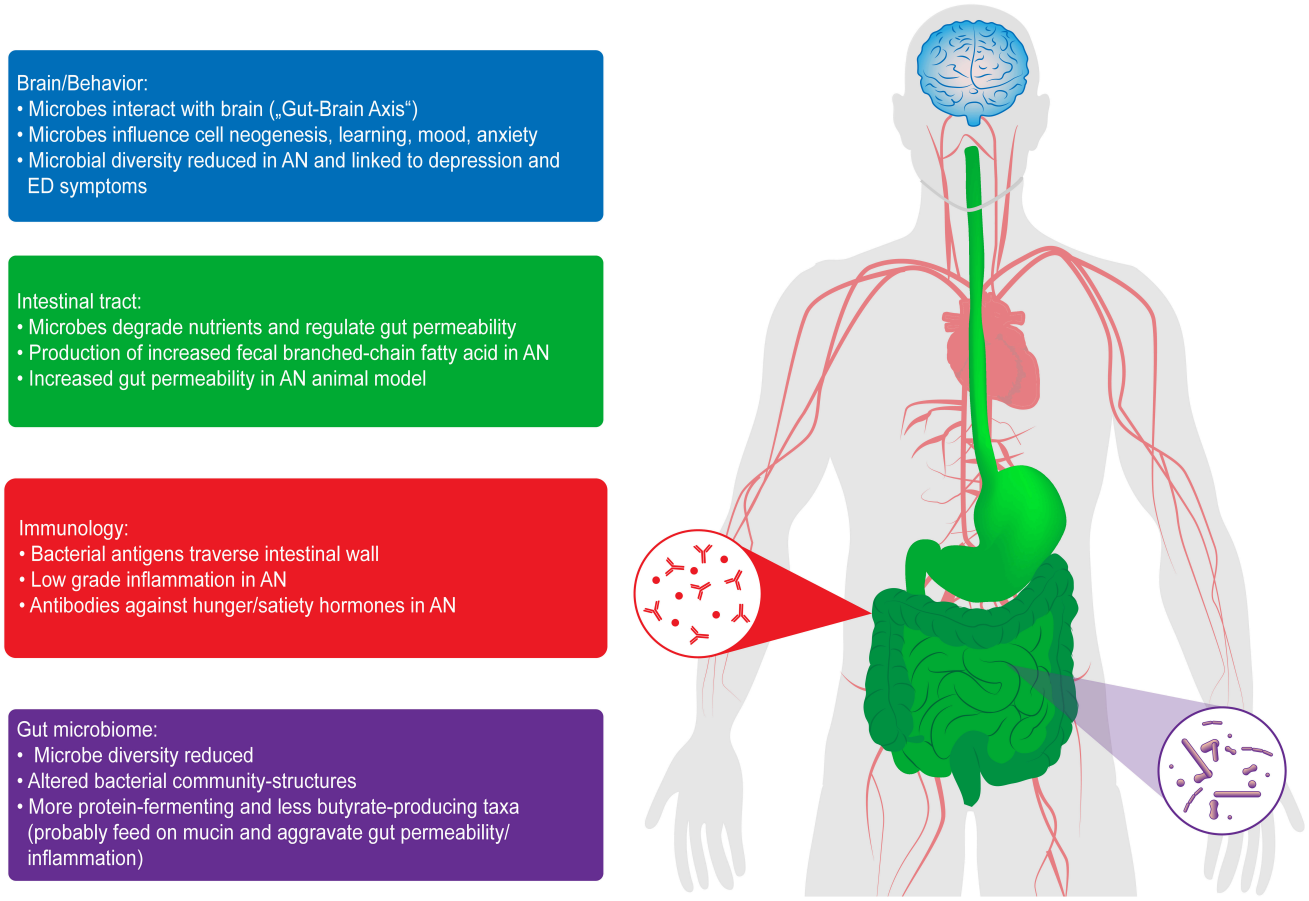
Interactions of the gut microbiome with the brain and behavior, in the intestinal tract and with immunology in patients with AN. ED, eating disorder [from Seitz et al. (18)].

Evidence is now growing that AN-induced starvation is associated with profound alterations of the gut microbiome (10–13), which is of critical interest given its important interactions with the host metabolism in terms of weight regulation, hormonal, immunologic and inflammatory processes, along with a direct influence on the brain and behavior (“gut-brain axis”) (14–18) (see Figure 1). AN is one of the most “exemplary” disorders for studying gut-brain interactions because no other mental disease exists in which nutrition and its changes, which are important factors influencing microbial growth in the intestine, play such a crucial role (19). Based on the DSM-5, we differentiate between two subtypes of AN, restrictive and binge/purging. While the restrictive subtype is characterized by fasting and often, but not always, physical hyperactivity, patients classified as the binge/purging subtype may engage in binging and purging (e.g., laxative abuse or self-induced vomiting), only binging (with intermittent periods of fasting or excessive exercising) or only purging. In an important study by Mack et al. (10) significant differences in the microbiome were found between the restrictive and the binge/purging subtypes. Moreover, we may speculate that iatrogenic changes in the diet implicated in the treatment of AN, and thus of the microbiome, might also impact the course of the disorder.

Each human individual has close to 500 out of approximately 1,000 different gut microbial species (20), yielding a highly personal composition. Many digestive functions are thought to have been “delegated” to the gut microbiome over the course of evolution. In this symbiotic relationship, certain gut microbes break down food to supply the host with essential vitamins, fatty acids and other nutrients that the host would otherwise be unable to extract. However, the role of the gut microbiome extends well beyond digestion: the community of microbes is in continuous exchange with the cells of the gut wall and beyond, informing our immune system and affecting intestinal permeability, hormones and inflammation (15). Mechanisms involved in the gut-brain axis that directly influence the brain and behavior are not well understood, but seem to include direct vagal nerve signaling, immune cell migration to the brain, antibodies, cytokines and hormones (15, 21, 22). Causal roles of microbiome alterations have been found in anxiety disorder, depression and reactions to stress (21), all of which are important symptoms or comorbidities of AN (1). In this review, we summarize the pertinent findings regarding these mechanisms of interaction between the microbiome and the host and how they can influence the pathophysiology and symptoms of AN.

## Hormones

AN is characterized by profound endocrine alterations (23). Gut microbes are influenced by hormones and neurotransmitters and in turn affect hormonal and neurotransmitter secretion. Consistent evidence of multiple pathways of bidirectional interaction [for a review, see Neuman et al. (15)], including microbes producing dopamine (24), serotonin (24), norepinephrine (25) and GABA (25), as well as insulin and blood sugar regulation (26), has been reported. Germ free (GF) mice, born and raised in completely sterile environments, have proven to be valuable sources of information. Compared to normally raised control animals, they exhibited a 25% increased TSH level (27), but decreased plasma catecholamine (25), and serotonin (28) levels. The satiety hormone leptin was associated with an increased abundance of *Bifidobacterium* and *Lactobacillus* and a decreased abundance of *Bacteroides* and *Prevotella*, while the hunger hormone ghrelin showed inverse relationships (29). Gut bacteria are thought to help regulate orexin release from neuroendocrine cells which in turn influences local neural communication in the gut as well as central nervous functions of hunger signaling (30). Patients with AN have increased ghrelin and orexin and reduced leptin and thyroxin levels, so hormonal interaction with the microbiome seems likely. Amenorrhea induced by a chronic estrogen deficit is a core symptom of AN. Estrogen has been shown to decrease bacterial virulence but enhance bacterial culture growth rates (24), so further interactions with the gut microbiome can be expected. Lastly, starvation in AN is characterized by increased serum, urinary and salivary cortisol levels (23). GF-mice also showed elevated corticosterone levels, indicating a stress modulating and potentially stress reducing role of the microbiome (31). Notably, the administration of *Lactobacillus* and *Bifidobacteria* decreased circulating corticoid hormone levels in both humans and rats (32), further emphasizing the causal role of the microbiome in stress regulation, which might suggest a useful therapeutic option.

## Energy Harvest/Body Weight

The microbiome plays a central role in the amount of energy harvested from a specific quantity of food, with important implications for body weight regulation. In 2005, it was discovered that compared with normal weight controls, overweight patients have an altered gut microbiome, which appears able to extract more energy from the same food (33). Furthermore, a causal role of the microbiome was demonstrated when stool transplanted from obese mice into GF-mice resulted in greater weight gain than stool from lean rodents (34). This finding was confirmed in humans when stool transferred before bariatric surgery caused rats to increase in fat mass, whereas stool transferred after surgery did not (35). Stool transfer from the malnourished child of twin children discordant for kwashiorkor in Malawi into GF-mice induced weight loss and malnutrition (36). Additionally, oral antibiotics measurably ameliorated the nutritional state of children from Malawi (37). The proportion of Bacteroidetes was found to be a potentially important factor, as their abundance was associated with body mass index in normal-, under-, and overweight patients (12). Notably, Bacteroidetes is decreased in acutely ill patients with AN and increased upon weight recovery (10, 11, 38). Mack et al. also showed a significant difference of the microbiome between the binge-purging and the restrictive subtypes of AN (10). Differences in the microbial species that extract energy from the same quantity of food could help explain why patients with the restrictive subtype require dramatically more calories to gain weight compared to patients with the binge-purging subtype (39).

## Intestinal Permeability

Elevated stress and cortisol levels increase gut permeability, increasing the number of digestion components traversing the gut wall barrier and entering intra- and extra-cellular spaces in the host (40). However, whether higher cortisol levels in patients with AN also cause increased gut permeability is currently being researched. Mörkl et al. (41) could not provide evidence of an increased intestinal permeability in an initial study in patients with AN using blood zonulin levels, and Monteleone et al. (42) found reduced permeability in the small intestine by studying lactulose/mannitol absorption. However, Jesus et al. (43) showed a “leaky gut” in the colon, including fewer tight junction proteins, reduced gastric wall thickness and increased colonic permeability using the Activity-Based Anorexia mouse model. Achamrah et al. (44) also found increased colonic permeability using FITC-Dextran in the same AN animal model, and our own animal research may further support these initial findings, showing reduced cryptal depth and decreased tight junction proteins in the colon of rats, but not in the small intestine (manuscript in preparation). The gut microbiome interacts with the intestinal wall and strongly influences its permeability and barrier function (45). The microbiome in patients with AN appears to shift toward an increase of mucin-degrading Firmicutes and Verrucomicrobia and away from the carbohydrate-degrading species Bacteroidetes (10, 11) (see below). This shift could increase digestion of the protective intestinal wall mucus and further weaken the intestinal wall barrier in the colon, allowing greater translocation of bacterial products and components (10, 43), which might trigger immune and inflammatory responses (46–48).

## AN—An Autoimmune Disorder?

In two recent meta-analyses, patients with AN showed a low-grade inflammatory state with increased TNF-alpha, IL-6, and IL-1-beta (49, 50). As certain Lactobacilli can reduce TNF-alpha, IL-6, and IL-8 (51), this approach may offer an interesting treatment option for reducing intestinal permeability and inflammation in patients with AN. Fetissov et al. (52) found that the translocation of bacteria and their subcomponents across the intestinal wall can also cause cross-reactive antibodies to form, which can bind to hunger and satiety hormones such as ghrelin or alpha-melanocyte stimulating hormone (alpha-MSH) (53), thus, altering food intake and weight regulation (54, 55). Moreover, increased levels of antibodies for a large number of hunger and satiety hormones including leptin, ghrelin, orexin, and alpha-MSH were found in patients with eating disorders, including patients with AN, and some of the antibodies even correlated with eating disorder symptom severity (54, 56). A potential mechanism could be that these antibodies protect these hormones from degradation, as could be evidenced for anti-ghrelin antibodies (55, 57). Auto-antibodies could thus be an important underlying phenomenon in AN and one mechanism by which the gut microbiome influences the brain and complex behaviors such as hunger and satiety (18). Notably, patients with AN also showed a generally increased proneness to autoimmune diseases in large Finnish and Swedish cohorts (58, 59). Specifically, the prevalence of gastrointestinal (OR 1.8) and endocrine autoimmune diseases (OR 2.4) was higher, whereas Crohn's disease showed the highest rate with an OR of 3.9. Patients with autoimmune diseases also had an increased risk of AN (60). Anecdotally, a Crohn's disease patient also affected by AN reported a significant improvement in AN symptoms after anti-TNF-alpha treatment, suggesting another link between autoimmune diseases and AN (61).

## Gut-Brain Axis

The gut microbiome has a significant effect on our development and the brain, starting at birth. Contact with different bacteria, depending on our mode of birth, i.e., vaginally or via C-section, and whether we were breastfed strongly influence which bacteria inhabit our gastrointestinal tract, with probably lifelong consequences for our immune system (62). The number of metabolic and neurologic diseases with altered gut microbiome and evidence for gut-brain interactions is constantly rising (63): In type 2 diabetes the microbiome appears to disturb the enteric nervous system via inflammatory processes, impairing its role in informing the hypothalamus of the nutritional state to properly control glucose entry in tissues (64, 65). In Parkinson's disease, α-Synuclein aggregates, the major neuropathologic marker of the disease, are detectable in the enteric nervous system prior to the brain, with a possible gut to brain prion-like transmission (66); abundance of Prevotellaceae is reduced by 80%, and abundance of Enterobacteriaceae was highly correlated with postural instability and gait difficulty (67). Stool from depressive patients, who often show increased inflammatory markers, was able to evoke depressive-like symptoms when introduced into antibiotics-treated rodents - but not the stool of healthy controls with no mood disorder (68). In 18 children with autism, who had a heavily altered gut microbiome, continued stool transplants from healthy subjects normalized the microbiome, reduced gastrointestinal symptoms by 80% and improved autistic symptoms for at least 8 weeks after treatment ended (69). Again, causal inferences can be made by studying GF-mice: they exhibit deviant morphologic and functional brain development (70) and different brain-derived neurotropic factor (BDNF) concentrations in the blood and the hippocampus (71), which influence neuron growth and protection as well as synapse formation and connectivity. Notably, patients with AN also showed reduced BDNF serum levels during semi-starvation, which were alleviated upon weight restoration (72). Antibiotics were shown to decrease BDNF levels in the hippocampus in GF-animals increasing levels of anxiety and thus indicating a direct link between the microbiome, behavior and the brain (73). This finding was further corroborated in a study with mice in which antibiotics were used to eradicate the gut microbiome. The result was decreased cell neurogenesis in the hippocampus with ensuing learning deficits, mediated by certain monocytes that could cross the blood-brain barrier. Probiotics, the application of potentially beneficial live bacteria (here, Bifidobacteria and *Lactobacillus*), were able to reverse both deficits (22). Patients with AN have been shown to suffer from significant brain volume loss, both in gray and white matter (74), associated with neuropsychological deficits and negative clinical prognosis (75). Here, our own group could also identify underlying impaired cell neogenesis in the AN animal model that was most strongly affecting astrocytes (76). We also found functional memory impairments (77). The neurotransmitter serotonin seems to be another important mediator between the microbiome and the brain. GF-mice showed a significantly higher level of serotonin metabolite 5-hydroxyindoleacetic acid [5-HIAA] in the hippocampus (78). The same metabolite was found to be reduced in the CSF of patients with AN during the acute phase, but it increased after weight recovery (79). The exact link between microbiota and brain changes in AN, however, remains to be elucidated.

## Initial Findings of AN

Initial studies analyzing altered microbiomes in patients with AN have reported heterogeneous results (14, 16, 18, 80). Cross-sectional studies involving between nine and 20 patients (12, 13, 38, 41, 81, 82), a longitudinal study with three patients (83) and combined cross-sectional studies with a longitudinal follow-up of up to 44 patients (10, 11) have been reported. Two studies found reduced alpha diversity (measuring the number of different species in the sample) (11, 41), whereas two others found changes in the same direction that were not significant (10, 82). Lower alpha diversity is commonly interpreted as disequilibrium of the microbial community, potentially decreasing its genetic pool and ability to react to disturbances. Reduced alpha diversity was also observed in patients with obesity and inflammatory bowel disease and has been correlated with heightened inflammatory levels and intestinal permeability (84). Mack et al. (10) showed a significant increase in alpha diversity during weight restoration, and Kleiman et al. (11) found a trend in the same direction. In Kleiman et al.'s cohort, alpha diversity in the starved state was correlated with eating disorders and depressive and anxious symptoms (11), highlighting another functional relevance of this finding. Increased beta diversity (measuring the dissimilarity of the microbiome between different individuals in the group) was found for two groups (10, 11), showing an increased variability in the AN sample. This diversity decreased after weight rehabilitation, but importantly, the microbiome in the patients with AN nevertheless more closely resembled their own microbiome before weight gain than that of the healthy controls (10). Firmicutes increased in abundance whereas Bacteroidetes decreased in two samples, as noted above (10, 11). The archaea *Methanobrevibacter smithii* increased in three samples (10, 81, 82), while the *Roseburia* species decreased in two samples (10, 82). *M. smithii* was inversely correlated with body mass index in one study (81). Its presence is typically interpreted as an adaptive mechanism to increase energy exploitation (10). Increased protein-degrading Firmicutes, as mentioned, could further induce mucin reduction and intestinal permeability. Decreased *Roseburia*, which feeds on carbohydrates and normally produces butyrate, could additionally increase intestinal permeability (85) and inflammation (86) due to a lack of the protective impact of this non-branched-chain fatty acid. Two studies showed heightened levels of branched-chain fatty acids. These digestive products of protein fermentation were previously found to increase PYY-production, a gastric peptide known to decrease appetite and increase depressive symptoms (87).

## Consequences for Future Research and Therapeutic Outlook

Research on the interaction of the human microbiome with its host is rapidly increasing for a variety of somatic and mental disorders (14, 21, 46, 84). In patients with AN, we require longitudinal observation studies that identify the role of the gut microbiome in energy utilization, appetite, gastrointestinal symptoms, neurocognition and learning, depression, anxiety, and clinical outcome. To clarify the pathophysiologic mechanisms, human and animal intervention studies that pay careful attention to determining causality are required. Furthermore, we must critically analyze current refeeding practices for patients with AN. As nutrition is a crucial influencing factor of the gut microbiome, we must question the provision of standard hospital food to patients without considering the patients' prior diets. The rapid change from typically low amounts of a fiber-rich, low-fat diet prior to admission to a high caloric, high fat and carbohydrate diet will affect the composition of their gut microbiome. The change from a non-animal- to an animal-based diet can tremendously impact the microbiome (19). However, patients who have often been on a vegan or vegetarian diet and who require oral supplements or nasogastric feeding typically receive cow milk-based products, with unclear consequences on their microbiome (14, 17). Most doctors, furthermore, do not know that 25% of all drugs (not only antibiotics) impact the gut microbiome (88). Thus, research on refeeding therapy for AN is urgently required to prevent causing iatrogenic harm to our patients (14).

Future additions to traditional AN therapy aiming to positively influence the gut microbiome could include nutritional interventions, food supplements, probiotics and prebiotics (dietary fibers favoring the growth of certain bacteria). The goals could be to increase the amount of energy harvested from the same quantity of food and to decrease gut permeability, inflammation and antibody formation, with the potential consequence of reducing depressive and anxious symptoms. Preclinical research has demonstrated promising results (45), and healthy controls have shown improved mood and cognitive function in a recent study (89, 90). A recent systematic review of 10 RCTs on the use of probiotics for anxiety and depression in patients found, despite methodological limitations because of the different bacterial strains applied, “preliminary evidence of psychological benefits” (91).

## Author Contributions

JS, BH-D, and JB: conception and design; JS, NS, and BH-D: literature search; MB, JB, and AD: critical evaluation of literature search results; JS and BH-D: draft; MB, JB, AD, NS, BH-D, and JS: revisions.

### Conflict of Interest Statement

The authors declare that the research was conducted in the absence of any commercial or financial relationships that could be construed as a potential conflict of interest.
